# Draft Genome Sequence of the Thermophile *Thermus filiformis* ATCC 43280, Producer of Carotenoid-(Di)glucoside-Branched Fatty Acid (Di)esters and Source of Hyperthermostable Enzymes of Biotechnological Interest

**DOI:** 10.1128/genomeA.00475-15

**Published:** 2015-05-14

**Authors:** Fernanda Mandelli, Brenda Oliveira Ramires, Matthew Brian Couger, Douglas A. A. Paixão, Cesar M. Camilo, Igor Polikarpov, Rolf Prade, Diego M. Riaño-Pachón, Fabio M. Squina

**Affiliations:** a Laboratório Nacional de Ciência e Tecnologia do Bioetanol (CTBE), Centro Nacional de Pesquisa em Energia e Materiais (CNPEM), Campinas, SP, Brazil; b Departamento de Ciência de Alimentos da Faculdade de Engenharia de Alimentos, UNICAMP, Campinas, SP, Brazil; c Department of Microbiology and Molecular Genetics, Oklahoma State University, Stillwater, Oklahoma, USA; d Instituto de Física de São Carlos, Universidade de São Paulo, São Carlos, SP, Brazil

## Abstract

Here, we present the draft genome sequence of *Thermus ﬁliformis* strain ATCC 43280, a thermophile bacterium capable of producing glycosylated carotenoids acylated with branched fatty acids and enzymes of biotechnological potential.

## GENOME ANNOUNCEMENT

*Thermus ﬁliformis* strain (ATCC 43280—obtained from the National Institute for Quality Control in Health, FIOCRUZ—Rio de Janeiro, Brazil) was first isolated from a hot spring in New Zealand (1). *T. ﬁliformis* forms long ﬁlaments consisting of chains of cells and can thus be morphologically distinguished from other *Thermus* strains (1). This strain is particularly interesting, as it was reported to produce thermozeaxanthins and thermobiszeaxanthins, which are carotenoid-(di)glucoside-branched fatty acid (di)esters (2). Carotenoids are pigments known for their ability to act as potent antioxidants, protecting cells and tissues from the damaging effects of reactive oxygen and nitrogen species. Besides carotenoid production, *T. ﬁliformis* can be considered a source of hyperthermostable enzymes of biotechnological interest.

Here we present the genome sequence of *T. filiformis* ATCC 43280. This genome was sequenced on the Illumina MiSeq sequencing system, generating 7,099,814 paired-end reads of 250 bp (insert size, 250 bp) and 2,124,227 mate-pair reads of 150 bp (insert size between 2 Kbp and 15 Kbp). Paired-end reads were preprocessed with Trimmomatic (3), and mate pairs with NextClip (4), resulting in 1,857,428 and 621,279 cleaned reads, respectively. The genome size was estimated to be 3.1 Mbp based on k-mer count statistics (5), with an estimated coverage of 580×. Chromosomal assembly was carried out with SPAdes v3.5.0 using an ensemble of k-mer values (K = [21, 33, 55, 77, 99, 127]). Presence of typical bacterial marker genes was assessed using Amphora2 (6). The resulting assembly has 40 scaffolds, with a total length of 2,386,081 bp and an *N*_50_ of 551,922 bp. The average G+C content of the genome is 68.9%, which is similar to related species, i.e., *Thermus thermophilus* HB8, GC of 69.5% (7); *T. oshimai* JL-2, GC of 68.6% (7); *T. scotoductus* SA-01, GC of 64.9% (8); *T. islandicus* DSM251543, GC of 68.3% (assembly, ATXJ00000000.1); *Thermus antranikianii* DSM12462, GC of 64.8% (assembly, GCA_000423905.1); and *Thermus aquaticus* Y51MC23, GC of 68.0% (assembly, ABVK00000000.2). Gene prediction was carried out with the NCBI Prokaryotic Genome Annotation Pipeline (9). A total of 2,405 genes were identified, of these, there are 2,211 protein-encoding genes, 6 rRNA genes, 47 tRNAs, and 9 pseudogenes. Gene content is similar to that of related species, i.e., *T. thermophilus* (2,035 genes), *T. oshimai* (2,205 genes), *T. scotoductus* (2,503 genes), *T. islandicus* (2,289 genes), *T. antranikianii* (2,225 genes), and *T. aquaticus* (2,593 genes). The current genome assembly provides a preliminary landscape of the genomic and metabolic capabilities of *T. filiformis*.

### Nucleotide sequence accession numbers. 

This whole-genome shotgun project has been deposited at DDBJ/EMBL/GenBank under the accession number JPSL00000000. The version described in this paper is version JPSL02000000.
